# Association Between Pediatric Obesity and Ocular Structural Parameters: A Cross-Sectional Study

**DOI:** 10.3390/children13070847

**Published:** 2026-06-23

**Authors:** Alev Koçkar, Ahmet Oran, Ayşe Nurcan Cebeci, Elvan Alper Şengül

**Affiliations:** 1 Department of Ophthalmology, Umraniye Training and Research Hospital, University of Health Sciences, Istanbul 34668, Turkey; drahmetoran@gmail.com; 2 Department of Pediatrics and Adolescent Medicine, Faculty of Medicine, Friedrich-Alexander-Universität Erlangen-Nürnberg, 91054 Erlangen, Germany; nurcancebeci@yahoo.com; 3 Department of Ophthalmology, Faculty of Medicine, Istanbul Bilim University, Istanbul 34920, Turkey; ealper_sengul@yahoo.com

**Keywords:** pediatric obesity, optical coherence tomography, retinal nerve fiber layer, ganglion cell complex, anterior chamber depth, ocular biometry, pediatric ophthalmology

## Abstract

Background/Objectives: To explore potential associations between pediatric obesity and retinal and anterior segment ocular structures using OCT and ocular biometry. This study was designed as an exploratory, hypothesis-generating analysis without a pre-specified primary endpoint; all findings should be interpreted accordingly. Methods: This retrospective cross-sectional study included 52 children (104 eyes): 27 obese children (body mass index (BMI) percentile ≥95%) and 25 healthy controls (BMI percentile 5–85%). Optical coherence tomography (OCT) and ocular biometry were used to assess retinal nerve fiber layer (RNFL), ganglion cell complex (GCC), focal loss volume (FLV), global loss volume (GLV), Early Treatment Macular Map 5 (EMM5), corneal parameters, axial length (AL), anterior chamber depth (ACD), and white-to-white corneal diameter (WTOW). Group comparisons and cluster-robust bootstrap regression adjusted for inter-eye dependency, age, and sex; Bonferroni correction was applied. Results: Obese children showed nominally higher GCC average thickness, RNFL, and EMM5 values and shallower ACD; however, no parameter survived Bonferroni correction. ACD showed the most internally consistent exploratory pattern (unadjusted *p* = 0.006; adjusted *p* = 0.018; Bonferroni *p* = 0.249); however, this finding did not survive Bonferroni correction and should not be interpreted as a confirmed association. Other corneal and biometric parameters were not significantly different. Conclusions: Pediatric obesity may be associated with subtle ocular structural variations, but all findings are exploratory and hypothesis-generating. Larger prospective, pre-registered studies are needed to determine whether pediatric obesity is associated with structural ocular changes.

## 1. Introduction

Obesity is a chronic metabolic disease that is increasingly prevalent in the pediatric population and can lead to a wide range of health problems. According to the World Health Organization, the global prevalence of overweight and obesity among children continues to rise each year, a trend associated with reduced physical activity, high-calorie dietary habits, and the widespread adoption of sedentary lifestyles driven by modern living conditions [1,2]. The significance of pediatric obesity extends beyond its well-known association with increased risks of diabetes, hypertension, and cardiovascular diseases in adulthood; it may also cause early organ involvement by inducing persistent systemic vascular and metabolic alterations [3].

Chronic inflammation, oxidative stress, and endothelial dysfunction associated with obesity can lead to structural changes, particularly in organs with rich microvascular networks [4,5]. The retina, characterized by its high metabolic activity and dense microvascular structure, is among the tissues most susceptible to these systemic effects. Evaluating changes in the retina and optic nerve head may not only offer a noninvasive indicator of systemic vascular health but also provide important clinical insights into the potential ocular consequences of obesity [6,7].

Regarding posterior segment changes, existing studies report heterogeneous but clinically important findings. Several groups have documented reduced peripapillary retinal nerve fiber layer (RNFL) thickness in obese children, with thinning correlating with body mass index (BMI) standard deviation score, insulin resistance, and systemic inflammatory markers [8,9]. Pacheco-Cervera et al. demonstrated RNFL thinning in children with severe obesity, suggesting that chronic low-grade inflammation may impair retinal nerve fiber integrity even in the pediatric age group [8]. In contrast, other studies found no significant overall RNFL difference, with only quadrant-specific or temporal outer macular variations [10,11], highlighting the inconsistency in the literature and the need for standardized, adequately powered studies. Macular thickness changes have also been reported: Öztürk et al. demonstrated significant subfield macular thickness variability in children with metabolic syndrome, consistent with the hypothesis that inner retinal layers are particularly sensitive to obesity-associated metabolic disturbances [12]. Regarding choroidal changes, Erşan et al. found narrower retinal arterioles, wider retinal venules, and thinner macular and subfoveal choroidal thickness in obese children using enhanced-depth imaging optical coherence tomography (OCT), suggesting early microvascular impairment [13]. Conversely, Bulus et al. reported increased subfoveal choroidal thickness positively correlated with BMI [14], while Topcu-Yilmaz et al. found that overall choroidal thickness was significantly lower in obese children compared with controls, with the most pronounced thinning observed in the subgroup without insulin resistance—suggesting that insulin resistance may paradoxically exert a protective or confounding effect on choroidal thickness in pediatric obesity [15], underscoring the complexity of choroidal responses to obesity and the potential modifying role of metabolic comorbidities. Retinal vessel caliber has also been shown to be altered in pre-adolescent obese children, with narrower arteriolar and wider venular diameters reflecting early arteriosclerotic and inflammatory processes [13,16,17]. Furthermore, retinal microvascular changes have been associated with metabolic risk factors even in pediatric patients, underscoring the potential of retinal imaging as a non-invasive window into systemic metabolic dysfunction in pediatric obesity [18].

With respect to anterior segment and biometric parameters, available evidence is more limited but growing. Elevated intraocular pressure (IOP) in obese children and adults has been consistently reported across multiple studies, likely reflecting increased episcleral venous pressure related to elevated intraabdominal pressure and adipose tissue accumulation [19]. Regarding anterior chamber depth (ACD), Gunes et al. demonstrated significantly shallower ACD in obese compared with healthy adults, with ACD negatively correlated with BMI, while axial length (AL) and central corneal thickness (CCT) remained similar between groups [20]. In a comprehensive pediatric study, Baran et al. evaluated IOP, RNFL, retinal ganglion cell layer, central macular thickness, and choroidal thickness using OCT in obese children and healthy controls, providing a broad multi-parameter assessment of obesity-related ocular changes in the pediatric age group [21]. Corneal parameters, including CCT, keratometry values, and corneal biomechanics, have also been assessed: Can et al. reported reduced corneal hysteresis in obese children, suggesting altered corneal viscoelastic properties potentially linked to systemic collagen or connective tissue changes associated with obesity [22]. These observations collectively suggest that pediatric obesity may be associated with a broad spectrum of subtle ocular structural alterations spanning both the posterior and anterior segments, though the evidence remains exploratory and heterogeneous [7,23,24].

Advances in OCT and ocular biometry technologies now allow highly accurate assessment of several ocular structures, including the RNFL, ganglion cell complex (GCC), corneal thickness, corneal curvature parameters, AL, and ACD. These parameters offer valuable information for understanding how pediatric obesity may affect both posterior and anterior segment components of the eye [25].

This study aimed to explore potential associations between pediatric obesity and retinal and anterior segment ocular structures, with the explicit goal of generating hypotheses for future confirmatory research. No primary endpoint was pre-specified; all 14 ocular parameters were treated equally as part of an exploratory, hypothesis-generating screen. To our knowledge, few studies have simultaneously evaluated both retinal and anterior segment structural parameters using OCT and ocular biometry in obese children. By comparing OCT and biometric parameters between obese and healthy children in an exploratory framework, we evaluated RNFL, GCC, pachymetry, AL, ACD, and corneal biometry measurements, with the goal of identifying potential associations between pediatric obesity and structural ocular parameters, not to establish causality.

## 2. Materials and Methods

### 2.1. Ethical Approval

The study was approved by the Clinical Research Ethics Committee of Istanbul Bilim University (decision no: 02.05.2017/58-26; document/reference no: 44140529/2017-60; date: 30 May 2017) and was conducted in accordance with the principles of the Declaration of Helsinki.

### 2.2. Study Population

This was a retrospective cross-sectional study in which clinical data, OCT measurements, and ocular biometry were collected from the medical records of patients who had previously attended the pediatric endocrinology and ophthalmology outpatient clinics. A total of 52 children were included in the study, and both eyes of each participant were evaluated, resulting in 104 eyes analyzed. Participants were classified according to BMI percentile values: 27 children with a BMI percentile ≥ 95% constituted the obese group, whereas 25 children with a BMI percentile between 5 and 85% formed the healthy control group. Written informed consent had been obtained from the parents of all participants prior to data collection at the time of their clinic visit. Children with systemic diseases, continuous medication use, a history of prematurity, prior ocular surgery, glaucoma or retinal pathology, or inadequate OCT signal quality were excluded from the study. Inclusion criteria were: age 6–18 years, BMI percentile ≥ 95% (obese group) or 5–85% (healthy control group), and availability of complete ophthalmic records. Exclusion criteria included any systemic disease (e.g., diabetes mellitus, hypertension, thyroid disorders), use of systemic or topical medications, history of prematurity (gestational age < 37 weeks), prior intraocular surgery, diagnosed glaucoma or retinal pathology, high refractive error (spherical equivalent beyond −6.00 or +4.00 D), and OCT scans with signal strength index < 7/10 or visible motion artifacts. These criteria were applied consistently across both groups.

### 2.3. Ophthalmologic Examination and Imaging Protocol

All participants underwent a standardized comprehensive ophthalmologic examination including best-corrected visual acuity assessment, slit-lamp biomicroscopy, IOP measurement using non-contact tonometry, and dilated fundus examination.

Retinal imaging was performed using a spectral-domain optical coherence tomography (SD-OCT) device (RTVue XR Avanti; Optovue Inc., Fremont, CA, USA; axial resolution: 5 µm; scanning speed: 70,000 A-scans/second; wavelength: 840 nm). Peripapillary RNFL thickness measurements were obtained using a 3.45 mm diameter circular scan centered on the optic disc. GCC analysis was performed using the macular GCC scan protocol covering a 7 × 7 mm area centered 1 mm temporal to the fovea. Average RNFL thickness, average GCC thickness, focal loss volume (FLV), and global loss volume (GLV) parameters were recorded. Macular thickness measurements were obtained using the EMM5 protocol.

Anterior segment and corneal topography measurements were acquired using the Orbscan II system (Bausch & Lomb, Rochester, NY, USA), which integrates slit-scanning technology with Placido disk-based corneal topography. The device provides pachymetric mapping and anterior/posterior corneal surface analysis. The following parameters were recorded: CCT, SimK, maximum and minimum keratometry values, K1 and K2 readings, and horizontal corneal diameter (white-to-white; WTOW).

AL and ACD were measured using a separate optical biometer (Lenstar LS900; Haag-Streit, Koeniz, Switzerland), which uses optical low-coherence reflectometry (OLCR) technology. The Orbscan II system, described above, was used exclusively for corneal topography and pachymetry measurements, and does not provide AL or ACD measurements. For each biometric parameter, at least three consecutive measurements were obtained, and the average value was used for statistical analysis. Only measurements with a signal strength index ≥ 7/10 and without motion artifacts were included. All measurements were performed by the same experienced technician to minimize interobserver variability.

In addition to ocular parameters, demographic and anthropometric data—including age, sex, height, weight, and BMI—were recorded. BMI was calculated as weight in kilograms divided by height in meters squared (kg/m^2^), and obesity status was determined according to age- and sex-adjusted percentile charts.

The analyzed ocular parameters comprised refractive, retinal, corneal, and biometric measurements. Refractive status was expressed as spherical equivalent (SE). Although SE was initially recorded, it was excluded from final analyses because of excessive missing-data imputation in the obese group. Specifically, SE data were missing for 14 of 27 obese participants (51.9%) and for 3 of 25 healthy controls (12.0%); the high proportion and asymmetric distribution of missing values in the obese group precluded valid group comparison and raised concerns about imputation bias. Retinal structural parameters included average peripapillary RNFL thickness and average GCC thickness, along with GCC FLV and GLV. Macular thickness was evaluated using the Early Treatment Macular Map 5 (EMM5) protocol. Corneal parameters included CCT, SimK, maximum and minimum corneal curvature values, as well as K1 and K2 readings. Ocular biometric measurements comprised AL, ACD, and white-to-white corneal diameter (WTOW).

## 3. Statistical Analysis

Data analyses were performed using IBM SPSS Statistics for MacOS, version 30.0 (IBM Corp., Armonk, NY, USA). Categorical variables were summarized as counts and percentages [*n* (%)], whereas continuous variables were presented as mean ± standard deviation (SD) for normally distributed data or median (minimum–maximum) for non-normally distributed data.

Normality was assessed using the Shapiro–Wilk test, which is recommended for sample sizes of fewer than 50 observations, together with skewness and kurtosis values. For comparisons between obese and healthy groups, Student’s *t*-test was used for normally distributed variables and the Mann–Whitney U test for non-normally distributed variables. Welch’s *t*-test was applied when variance homogeneity assumptions were violated. Categorical variables were compared using the chi-square test.

Subgroup analyses according to age and sex were performed using the Kruskal–Wallis test, followed by Bonferroni-corrected pairwise post hoc comparisons when overall significance was detected.

Effect sizes were calculated to facilitate interpretation of the magnitude of group differences. Cohen’s d was reported for parametric comparisons and rank-biserial correlation coefficient (r) for non-parametric comparisons.

The study includes both eyes from each participant, which raises the issue of non-independence of observations. Initial descriptive group comparisons (descriptive statistics and Mann–Whitney U/*t*-tests) were performed at the eye level, as this reflects the full dataset; however, we acknowledge that this approach may yield overly optimistic *p*-values if inter-eye correlation is not accounted for. To address this, cluster-robust bootstrap linear regression models (1000 bootstrap iterations; cluster = patient identity) were additionally constructed as the primary inferential analysis to account for inter-eye dependency. These models treat the patient as the unit of inference, correctly accounting for the correlation between fellow eyes. Obesity group was entered as the primary predictor, while age and sex were included as covariates. All *p*-values from the cluster-robust regression models are considered the primary inferential results; the descriptive eye-level comparisons are provided as supplementary information only.

**Figure 1:**
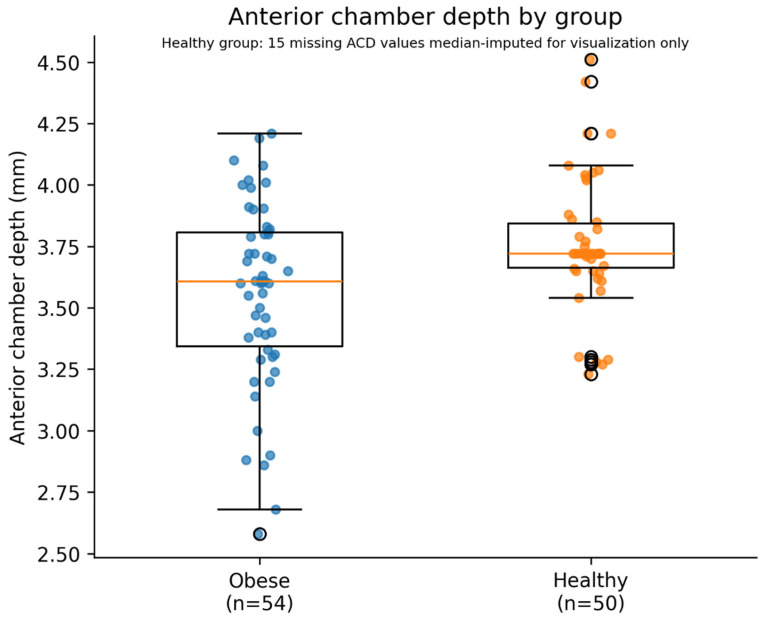
Distribution of anterior chamber depth measurements in obese and healthy children. Boxplots demonstrate the distribution of ACD values in obese and healthy participants. Individual eye measurements are overlaid. Obese children demonstrated relatively shallower ACD values compared with healthy controls. Median-imputed values were displayed for visualization only and were not included in any inferential analysis.

Because this was a retrospective exploratory study based on available clinical records, no formal a priori sample size calculation was performed. The available sample size was considered sufficient to detect only large between-group effects. Therefore, the study should be interpreted as exploratory and hypothesis-generating rather than confirmatory. This study was exploratory in design; no primary endpoint was pre-specified, and all 14 ocular parameters were treated equally as part of a hypothesis-generating screen. No finding should therefore be considered confirmatory. To reduce the risk of type I error arising from multiple simultaneous comparisons, Bonferroni correction was applied across the 14 ocular parameters evaluated in the study, yielding an adjusted significance threshold of *p* < 0.0036 (0.05/14). Both raw and Bonferroni-adjusted *p*-values are reported. Findings that were significant only before correction were interpreted as exploratory and hypothesis-generating. Regarding ACD: ACD was not successfully measured in 15 of 25 healthy control participants (60.0%) and in 0 of 27 obese participants, due to the optical biometer being unable to obtain a reliable ACD signal in these individuals (predominantly younger or less cooperative children in whom adequate fixation could not be maintained during the measurement). These missing values were not imputed in any statistical analysis; all inferential analyses for ACD (group comparisons, regression models) were performed using complete cases only (10 healthy control participants with valid ACD measurements, corresponding to the available complete-case healthy-eye dataset, vs. all 27 obese participants/54 obese eyes). The median imputation indicated in Figure 1 was applied solely for visualization purposes and was clearly labeled as such. Given the high proportion of missing ACD values in the healthy group, the ACD findings should be interpreted with particular caution, as the complete-case sample of healthy children may not be representative of the full control group. A sensitivity analysis restricted to children older than 10 years—in whom measurement success was higher—yielded directionally consistent results.

## 4. Results

### 4.1. Demographic and Clinical Characteristics

A total of 52 children were included (27 obese, 25 healthy controls), yielding 104 eyes. Mean age was 12.74 ± 3.04 years in the obese group and 10.76 ± 3.03 years in healthy controls (*p* = 0.023). This statistically significant age difference necessitated inclusion of age as a covariate in all regression analyses. Sex distribution did not differ significantly between groups (obese: 48.1% female; healthy: 56.0% female; *p* > 0.05). Mean BMI was 31.27 ± 5.52 kg/m^2^ in obese and 18.42 ± 2.51 kg/m^2^ in healthy controls (*p* < 0.001). Age-matching was not performed at the study design stage because this was a retrospective study based on available clinical records; groups were assembled according to BMI classification only.

### 4.2. Group Comparison: Obese vs. Healthy Children (Table 1)

Retinal parameters. GCC average thickness was higher in obese children (95.73 ± 7.20 µm vs. 92.23 ± 6.11 µm; *p* = 0.010, d = 0.524). GCC FLV was lower in the obese group (0.11% vs. 0.29%; *p* = 0.025). GCC GLV was also lower in obese children (2.76% vs. 4.07%; *p* = 0.015). RNFL average thickness was marginally higher in obese participants (107.47 µm vs. 103.85 µm; *p* = 0.048). EMM5 was significantly greater in the obese group (257.50 µm vs. 243.50 µm; *p* = 0.010). After Bonferroni correction for 14 simultaneous comparisons (α = 0.0036), none of these retinal parameters retained statistical significance (all adjusted *p* > 0.14), indicating that these findings should be interpreted as exploratory. The uncorrected *p*-values reported above are provided for descriptive completeness only; the Bonferroni-corrected results represent the primary inferential findings.

**Table 1:** Comparison of ocular structural parameters between obese and healthy children. Raw *p*-values and Bonferroni-adjusted *p*-values (*p*[Bonf]) are shown separately. All findings are exploratory; no parameter survived multiple-comparison correction.

Parameter	Obese (n = 54 Eyes)	Healthy (n = 50 Eyes)	*p* (Raw)	*p* (Bonf)	Test	Effect Size
GCC avg thickness (µm)	95.73 ± 7.20	92.23 ± 6.11	0.01	0.139	*t*-test	d = 0.524
GCC FLV (%)	0.11 (0.00–2.58)	0.29 (0.00–3.68)	0.025	0.349	MWU	r = 0.258
GCC GLV (%)	2.76 (0.07–17.56)	4.07 (0.08–18.15)	0.015	0.215	MWU	r = 0.279
RNFL avg thickness (µm)	107.47 (90.96–138.38)	103.85 (80.27–141.81)	0.048	0.666	MWU	r = 0.227
EMM5 (µm)	257.50 (217–309)	243.50 (216–279)	0.01	0.145	MWU	r = 0.303
Pachymetry (µm)	562.00 (485–649)	568.50 (507–638)	0.54	1	MWU	r = 0.070
Sim K’s (D)	−0.80 (−4.60–−0.10)	−0.75 (−1.80–−0.20)	0.623	1	MWU	r = 0.056
Max K (D)	43.45 (41.70–49.10)	43.75 (41.80–47.30)	0.417	1	MWU	r = 0.093
Min K (D)	42.75 (40.20–45.30)	42.70 (40.40–46.60)	0.99	1	MWU	r = 0.002
Axial length (mm)	23.76 ± 0.88	23.61 ± 1.07	0.439	1	*t*-test	d = 0.159
ACD (mm)	3.56 ± 0.37	3.78 ± 0.31	0.006	0.249	*t*-test	d = 0.629
K1 (mm)	7.85 ± 0.20	7.83 ± 0.24	0.695	1	*t*-test	d = 0.080
K2 (mm)	7.70 (6.93–7.98)	7.71 (7.06–8.09)	0.943	1	MWU	r = 0.009
WTOW (mm)	11.97 ± 0.38	11.96 ± 0.44	0.965	1	*t*-test	d = 0.009

Bonferroni correction was applied for 14 simultaneous comparisons (adjusted significance threshold: *p* < 0.0036). Normally distributed variables are presented as mean ± SD, whereas non-normally distributed variables are presented as median (min–max). Effect sizes are reported as Cohen’s d for parametric tests and rank-biserial correlation coefficients (r) for non-parametric tests. GCC = ganglion cell complex; FLV = focal loss volume; GLV = global loss volume; RNFL = retinal nerve fiber layer; EMM5 = Early Treatment Macular Map 5; AL = axial length; ACD = anterior chamber depth; WTOW = white-to-white corneal diameter. Spherical equivalent (SE) was excluded from final analyses.

Anterior chamber depth. ACD was shallower in obese children (3.56 ± 0.37 mm vs. 3.78 ± 0.31 mm; *p* = 0.006, d = 0.629), but this finding did not survive Bonferroni correction (adjusted *p* = 0.249). As exploratory context, the direction of the difference was consistent in the cluster-robust bootstrap regression analysis (*p* = 0.018, adjusted for age and sex); however, given that this finding did not meet the corrected significance threshold, it should be regarded as an exploratory signal warranting further investigation rather than a robust or confirmatory result (Figure 1).

No significant differences were detected in pachymetry, Sim K’s, Max K, Min K, AL, K1, K2, or WTOW (all raw *p* > 0.40; all adjusted *p* = 1.000), suggesting that, within the sample studied, fundamental corneal structure and globe dimensions did not differ significantly between groups; however, the small sample size limits the strength of this negative finding.

### 4.3. Subgroup Analysis by Age Group (Table 2)

Participants were stratified into four age-based subgroups: Obese ≤ 12 years (n = 11, 22 eyes), Obese > 12 years (n = 16, 32 eyes), Healthy ≤ 12 years (n = 18, 36 eyes), and Healthy > 12 years (n = 7, 14 eyes). Kruskal–Wallis tests were applied. After Bonferroni correction, no parameter reached the adjusted significance threshold (α = 0.0036).

At the uncorrected level, GCC average thickness (*p* = 0.021), GCC GLV (*p* = 0.027), RNFL average thickness (*p* = 0.044), and ACD (*p* = 0.031) showed nominally significant differences across age subgroups. Post hoc Bonferroni pairwise analyses identified the Obese >12 years vs. Healthy >12 years contrast (groups 2–4) as the primary driver for GCC average thickness, GCC GLV, RNFL, and ACD. These patterns are exploratory and require replication in larger samples.

**Table 2:** Age-stratified comparison of ocular parameters in obese and healthy children.

Parameter	Obese ≤ 12 yr (n = 11)	Obese > 12 yr (n = 16)	Healthy ≤ 12 yr (n = 18)	Healthy > 12 yr (n = 7)	*p* (Raw)	*p* (Bonf)	Post Hoc
GCC AV TH (µm)	96.22 (77–120)	96.88 (83–109)	92.39 (84–105)	87.81 (77–103)	0.021	0.288	2–4
GCC FLV (%)	0.09 (0.00–2.58)	0.16 (0.00–1.96)	0.28 (0.00–3.68)	0.41 (0.00–3.18)	0.096	1	—
GCC GLV (%)	3.05 (0.07–17.56)	2.57 (0.07–13.12)	3.88 (0.08–11.74)	7.88 (1.64–18.15)	0.027	0.378	2–4
RNFL AVG (µm)	104.40 (94–121)	109.37 (91–138)	105.35 (93–142)	103.81 (80–113)	0.044	0.614	2–4
EMM5 (µm)	254 (224–278)	258 (217–309)	239.50 (216–276)	248.50 (217–279)	0.065	0.915	—
Pachymetry (µm)	563 (510–604)	561 (485–649)	573 (520–638)	538 (507–612)	0.508	1	—
Sim K’s (D)	−0.80 (−4.60–−0.10)	−0.85 (−3.80–−0.20)	−0.70 (−1.80–−0.20)	−0.85 (−1.50–−0.40)	0.961	1	—
Max K (D)	43.35 (41.80–48.60)	43.50 (41.70–49.10)	43.75 (41.80–47.30)	43.95 (42.30–45.00)	0.596	1	—
Min K (D)	42.65 (40.60–44.10)	42.75 (40.20–45.30)	42.70 (40.40–46.60)	42.80 (41.50–44.50)	0.952	1	—
AL (mm)	23.65 (22.14–25.12)	23.64 (22.64–26.11)	23.43 (21.93–24.89)	24.04 (22.26–25.66)	0.063	0.883	—
ACD (mm)	3.66 (2.58–4.10)	3.60 (2.86–4.21)	3.71 (3.23–4.21)	3.94 (3.54–4.51)	0.031	0.429	2–4
K1 (mm)	7.86 (7.64–8.17)	7.85 (7.36–8.19)	7.88 (7.30–8.25)	7.86 (7.46–8.20)	0.84	1	—
K2 (mm)	7.73 (6.97–7.98)	7.69 (6.93–7.94)	7.73 (7.17–8.09)	7.69 (7.06–8.07)	0.75	1	—
WTOW (mm)	11.95 (11.10–12.34)	11.93 (11.04–12.83)	11.96 (11.02–12.66)	12.09 (11.13–12.60)	0.856	1	—

Kruskal–Wallis test. Bonferroni correction was applied for 14 simultaneous comparisons (adjusted significance threshold: *p* < 0.0036). Groups: 1 = Obese ≤ 12 years; 2 = Obese > 12 years; 3 = Healthy ≤ 12 years; 4 = Healthy > 12 years. Data are presented as median (min–max). No parameter remained statistically significant after Bonferroni correction. GCC = ganglion cell complex; FLV = focal loss volume; GLV = global loss volume; RNFL = retinal nerve fiber layer; EMM5 = Early Treatment Macular Map 5; AL = axial length; ACD = anterior chamber depth; WTOW = white-to-white corneal diameter.

### 4.4. Subgroup Analysis by Sex (Table 3)

Participants were grouped into: Obese Female (n = 13, 26 eyes), Obese Male (n = 14, 28 eyes), Healthy Female (n = 14, 28 eyes), and Healthy Male (n = 11, 22 eyes).

**Table 3:** Sex-stratified comparison of retinal, corneal, and biometric ocular parameters in obese and healthy children.

Parameter	Obese Female (n = 13)	Obese Male (n = 14)	Healthy Female (n = 14)	Healthy Male (n = 11)	*p* (Raw)	*p* (Bonf)	Post hoc
GCC AV TH (µm)	97.50 (83–120)	95.99 (77–109)	92.77 (77–103)	92.21 (82–105)	0.048	0.671	—
GCC FLV (%)	0.34 (0.00–1.95)	0.08 (0.00–2.58)	0.41 (0.00–1.58)	0.20 (0.00–3.68)	0.014	0.198	2–3
GCC GLV (%)	3.12 (0.07–13.12)	2.51 (0.07–17.56)	3.81 (1.64–18.15)	4.83 (0.08–14.80)	0.105	1	—
RNFL AVG (µm)	109.58 (91–121)	106.96 (94–138)	103.81 (80–142)	105.35 (86–139)	0.22	1	—
EMM5 (µm)	259 (217–309)	254.50 (224–288)	235.50 (216–279)	248 (220–276)	0.031	0.436	—
Pachymetry (µm)	558.50 (485–619)	569.50 (506–649)	538 (507–612)	598.50 (566–638)	<0.001	<0.001	1–4; 2–3; 2–4; 3–4
Sim K’s (D)	−0.80 (−1.70–−0.10)	−0.85 (−4.60–−0.10)	−0.65 (−1.50–−0.40)	−1.15 (−1.80–−0.20)	0.234	1	—
Max K (D)	43.70 (41.70–45.10)	43.25 (41.80–49.10)	43.75 (41.80–47.30)	43.95 (42.00–44.50)	0.838	1	—
Min K (D)	42.90 (40.20–44.30)	42.50 (40.80–45.30)	42.80 (40.40–46.60)	42.65 (41.20–43.70)	0.469	1	—
AL (mm)	23.11 (22.14–25.55)	24.03 (23.00–26.11)	23.82 (21.99–25.52)	23.56 (21.93–25.66)	0.018	0.246	1–2
ACD (mm)	3.60 (2.86–4.10)	3.64 (2.58–4.21)	3.73 (3.23–4.06)	3.72 (3.28–4.51)	0.072	1	—
K1 (mm)	7.81 (7.36–8.17)	7.95 (7.44–8.19)	7.80 (7.30–8.20)	7.87 (7.49–8.25)	0.296	1	—
K2 (mm)	7.67 (7.22–7.98)	7.82 (6.93–7.97)	7.70 (7.06–8.07)	7.73 (7.39–8.09)	0.725	1	—
WTOW (mm)	11.86 (11.10–12.59)	12.02 (11.04–12.83)	12.07 (11.38–12.60)	11.89 (11.02–12.66)	0.61	1	—

Kruskal–Wallis test. Bonferroni correction was applied for 14 simultaneous comparisons (adjusted significance threshold: *p* < 0.0036). Groups: 1 = Obese Female; 2 = Obese Male; 3 = Healthy Female; 4 = Healthy Male. Data are presented as median (min–max). Only pachymetry remained statistically significant after Bonferroni correction. GCC = ganglion cell complex; FLV = focal loss volume; GLV = global loss volume; RNFL = retinal nerve fiber layer; EMM5 = Early Treatment Macular Map 5; AL = axial length; ACD = anterior chamber depth; WTOW = white-to-white corneal diameter. Spherical equivalent (SE) was excluded from final analyses.

Pachymetry was the only parameter to survive Bonferroni correction (raw *p* < 0.001; adjusted *p* < 0.001). Multiple post hoc pairwise contrasts were significant (1–4, 2–3, 2–4, 3–4), reflecting sex-related variation in corneal thickness rather than an obesity-specific effect. Healthy Males showed the highest pachymetry (598.50 µm), consistent with published sex norms.

At the uncorrected level, GCC average thickness (*p* = 0.048), GCC FLV (*p* = 0.014), EMM5 (*p* = 0.031), and AL (*p* = 0.018) showed nominally significant sex-group differences. None survived Bonferroni correction. The AL difference (post hoc: 1–2) reflects normal sex-based globe size variation rather than an obesity effect. These findings are considered exploratory.

### 4.5. Cluster-Robust Bootstrap Regression Analysis (Table 4)

Cluster-robust bootstrap linear regression (1000 iterations; cluster = patient identity) was applied to adjust for inter-eye dependency and confounding by age and sex. Obesity group, age (continuous), and sex were entered as fixed predictors.

**Table 4:** Cluster-robust bootstrap linear regression analysis of the association between obesity and ocular parameters.

Parameter	β (Obesity)	95% CI (Bootstrap)	*p* (Raw)	*p* (Bonf)	Interpretation
ACD (mm)	−0.255	[−0.465, −0.044]	0.018	0.249	Exploratory only—raw *p* significant but does not survive Bonferroni correction; should not be interpreted as a confirmed association
GCC AV TH (µm)	3.95	[−0.302, +8.201]	0.069	0.961	Non-significant after adjustment
EMM5 (µm)	10.45	[−0.427, +21.328]	0.06	0.836	Non-significant after adjustment
RNFL (µm)	4.911	[−1.086, +10.907]	0.109	1	Non-significant
GCC FLV (%)	−0.229	[−0.540, +0.082]	0.149	1	Non-significant
Pachymetry (µm)	−11.973	[−29.001, +5.054]	0.168	1	Non-significant
AL (mm)	−0.052	[−0.635, +0.531]	0.862	1	Non-significant

Cluster-robust bootstrap linear regression (1000 iterations; cluster = patient ID). Fixed effects included obesity group, age (years), and sex. β = regression coefficient for obesity group (1 = obese, 0 = healthy). Bonferroni correction was applied for 14 simultaneous comparisons (adjusted significance threshold: *p* < 0.0036). GCC = ganglion cell complex; FLV = focal loss volume; RNFL = retinal nerve fiber layer; EMM5 = Early Treatment Macular Map 5; AL = axial length; ACD = anterior chamber depth; CI = confidence interval. Spherical equivalent (SE) was excluded from final analyses.

ACD showed the most internally consistent exploratory pattern across analyses (β = −0.255 mm; 95% CI: −0.465, −0.044; *p* = 0.018 in adjusted analysis; *p* = 0.006, d = 0.629 in unadjusted comparison); however, it did not survive Bonferroni correction (adjusted *p* = 0.249) and should be considered an exploratory signal only. No confirmatory conclusions can be drawn from these data.

GCC average thickness (β = +3.950 µm; *p* = 0.069) and EMM5 (β = +10.450 µm; *p* = 0.060) showed borderline associations after covariate adjustment, suggesting partial attenuation by age and sex. These findings are hypothesis-generating and should be investigated in larger prospective studies with pre-registered primary outcomes.

**Figure 2:**
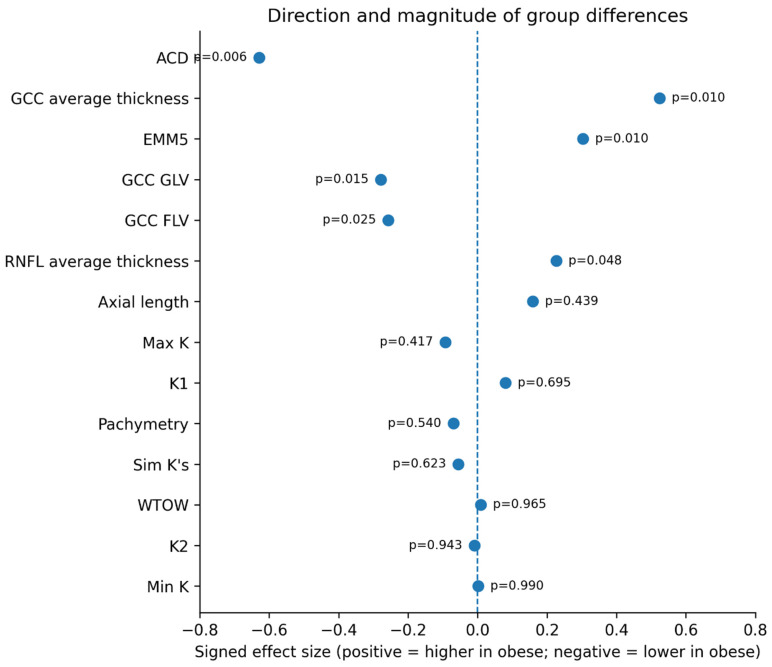
Magnitude and direction of obesity-related differences across ocular parameters. Forest-style effect size plot demonstrating the magnitude and direction of group differences between obese and healthy children across evaluated ocular parameters. Positive values indicate higher measurements in obese participants, whereas negative values indicate lower measurements. Values represent Cohen’s d or rank-biserial correlation coefficients derived from univariate analyses.

All remaining parameters showed no significant independent association with obesity in the regression analyses (all *p* > 0.10) (Figure 2).

## 5. Discussion

In this study, pediatric obesity was associated with nominal differences in several retinal and anterior segment ocular parameters, including GCC average thickness, EMM5, and ACD. However, none of these findings remained statistically significant after Bonferroni correction. Among all evaluated parameters, ACD showed the most internally consistent exploratory pattern across both unadjusted and adjusted analyses; however, it did not survive Bonferroni correction and should not be interpreted as a confirmatory obesity-related finding. In contrast, no substantial differences were observed in RNFL thickness, corneal curvature parameters, pachymetry, or AL measurements. Overall, these findings suggest possible subtle ocular structural variations in pediatric obesity, although the results should be interpreted cautiously given the exploratory nature of the analyses and the absence of Bonferroni-surviving findings.

Pediatric obesity is known to induce vascular and microvascular organ damage through inflammation, oxidative stress, and endothelial dysfunction. The retina, with its dense microvascular network and high metabolic activity, is among the tissues most susceptible to these systemic effects. As highlighted in the review by Dezor-Garus et al., obese children may demonstrate alterations in retinal vessel calibers, RNFL and macular thickness, and choroidal thickness, although considerable heterogeneity exists across studies [7]. The absence of a significant difference in RNFL average thickness between obese and healthy participants in our study aligns with findings reported by Koca et al., who observed comparable RNFL thicknesses in obese children, with only quadrant-specific variations favoring thicker measurements in controls [26]. Similarly, in the optical coherence tomography angiography (OCTA)-based study by Kurtul et al., no significant RNFL differences were found between obese and healthy children, while greater foveal retinal thickness and increased vascular density parameters were reported in obese individuals [27]. The absence of a significant overall RNFL difference in our study is further consistent with the findings of Özen et al., who reported a non-significant general decrease in RNFL thickness in obese children, with BMI standard deviation score (SDS) negatively correlating with RNFL in both groups [11]. In contrast, Karti et al. found significant thinning in all quadrants in a larger cohort [9], while Pacheco-Cervera et al. reported RNFL thinning specifically in children with severe obesity, suggesting that the degree of obesity may modulate the retinal response [8]. The observed discrepancies across studies likely reflect differences in sample size, obesity severity, OCT device, and the specific quadrants analyzed. Taken together, the heterogeneous RNFL findings in the literature—including our null result—underscore the need for larger, standardized, prospective studies with pre-specified RNFL as the primary endpoint. Thus, our RNFL findings are consistent with a substantial portion of the existing literature.

The observation that GCC average thickness was increased in obese children may indicate that inner retinal layers are more sensitive to obesity-related metabolic changes than the RNFL. Some studies have reported increased macular thickness and foveal vascular density in obese children, and the work by Kurtul et al. specifically demonstrated increased foveal retinal thickness and greater choriocapillaris flow area [27]. The elevated EMM5 values in our cohort support this notion. Furthermore, the study by Öztürk et al., which found significant variations in macular thickness and subfield measurements among children with metabolic syndrome, reinforces the concept that obesity and associated metabolic disturbances may exert structural effects on macular architecture, consistent with the increased GCC values observed in our study [12]. Dezor-Garus et al. also suggested that inner retinal layer thickening due to hyperperfusion in obese children may later evolve into thinning during adulthood due to chronic inflammation and microvascular damage [7].

Among anterior segment parameters, the shallower ACD observed in obese children represented the most internally consistent exploratory signal across the performed analyses; however, it did not survive Bonferroni correction (adjusted *p* = 0.249) and no confirmatory conclusion can be drawn from these data. Although the number of studies examining anterior segment biometry in obese children is limited, some reports evaluating intraocular pressure and ocular pulse amplitude have shown increased IOP and reduced ocular pulse amplitude in obese children [19]. Importantly, Gunes et al. demonstrated significantly shallower ACD in obese adults, with ACD negatively correlated with BMI [20], a finding directionally consistent with our observation in the pediatric population. Similarly, Baran et al. reported a comprehensive OCT-based assessment in obese children and healthy controls, confirming elevated IOP alongside structural OCT changes, underscoring the multidimensional impact of obesity on anterior segment physiology [21]. Given the high proportion of missing ACD data in the healthy control group (60%, 15/25 participants), these directionally consistent findings should be interpreted with particular caution and regarded as preliminary only. Regarding corneal biomechanics, Can et al. found reduced corneal hysteresis in obese children [22], suggesting that even in the absence of keratometric changes, obesity may affect corneal viscoelastic properties. The recent study by Uzun et al. further confirmed elevated IOP and central corneal thickness in overweight and obese children using Scheimpflug imaging and specular microscopy [24]. Taken together, the shallower ACD observed in our study warrants targeted investigation in future pre-registered studies with adequate statistical power, but should not be interpreted as an established obesity-related ocular finding.

Age-stratified analyses should be interpreted with great caution, as the subgroups—particularly healthy children older than 12 years—contained very few participants, substantially limiting statistical reliability and increasing the risk of chance findings. These analyses are therefore presented as purely exploratory and hypothesis-generating. With this important caveat in mind, the nominal differences observed in GCC average thickness, GCC GLV, RNFL, and ACD appeared to be driven primarily by the contrast between obese and healthy children older than 12 years (post hoc groups 2–4). In a broader context, the study by Öztürk et al., which reported associations between macular retinal parameters and anthropometric, metabolic, and inflammatory markers in children with metabolic syndrome, supports the concept that obesity-related metabolic disturbances may be linked to macular structural variability [12]. However, given the very small subgroup sizes and the absence of Bonferroni-surviving findings, no biological or confirmatory conclusions can be drawn from these subgroup data.

Sex-based analyses revealed notably greater differences in certain parameters among obese girls compared with healthy girls and/or healthy boys. This may reflect potential interactions between pubertal hormonal changes and metabolic processes that could influence certain ocular findings in female children. Dezor-Garus et al. also discussed the potential influence of puberty on the severity of obesity-related retinal changes [7]. However, given the limited literature on sex-specific ocular biometry variations in obese children, further studies with larger sample sizes are required to validate these findings.

The absence of significant differences in corneal thickness, SimK, K1–K2, maximum and minimum K values indicates that obesity may not substantially affect corneal stromal structure. Similar observations were reported by Hazar et al., who found largely comparable corneal thickness parameters in obese and healthy children, noting a relative resistance of corneal tissue compared with retinal layers [28]. Uzun et al. similarly found no significant differences in most anterior chamber parameters beyond IOP and CCT in a large cohort of obese and overweight children [24], while Can et al. reported that corneal hysteresis was significantly lower in obese children, suggesting subtle biomechanical alterations despite preserved keratometric values [22]. These findings collectively suggest that while gross corneal morphology and curvature may be relatively preserved in pediatric obesity, functional and biomechanical properties may be subclinically affected. AL was also similar between groups in our study, suggesting that pediatric obesity may not substantially influence overall globe elongation. Previous studies evaluating AL in obese children have yielded inconsistent results, with several reporting no significant differences between obese and healthy participants, consistent with our findings. Overall, the absence of significant corneal curvature and axial length differences suggests that fundamental globe morphology may be relatively resistant to obesity-related changes in childhood, at least within the range of obesity severity represented in our cohort.

From a clinical perspective, the present findings contribute to a growing body of evidence supporting the potential role of OCT as a non-invasive biomarker for obesity-related microvascular and structural alterations in children. OCT provides rapid, repeatable, and radiation-free imaging of retinal, macular, and anterior segment structures, making it particularly well-suited for use in pediatric clinical settings. The retina, as an accessible extension of the central nervous system with a rich microvascular supply, may serve as a unique window into systemic metabolic dysfunction: obesity-associated chronic inflammation, oxidative stress, and endothelial dysfunction may manifest as measurable structural changes in retinal layers before overt systemic complications develop [4,5,7]. The observation in our study that GCC average thickness and EMM5 showed borderline associations with obesity after covariate adjustment—even without surviving multiple-comparison correction—is consistent with the hypothesis that inner retinal layers may be early and sensitive targets of obesity-related metabolic disturbance. Erşan et al. demonstrated that arteriolar narrowing, venular widening, and choroidal thinning were detectable in obese children using enhanced-depth imaging OCT, suggesting that retinal microvascular changes precede clinically apparent disease [13]. Similarly, Celik et al. confirmed retrobulbar hemodynamic changes and microvascular alterations in obese children using Doppler and OCTA methods [23]. Liccardo et al. further established that retinal microvascular abnormalities in obese pediatric patients with NAFLD correlate with systemic metabolic risk factors, reinforcing the concept that retinal imaging may serve as an early, non-invasive metabolic risk indicator in pediatric obesity [18]. Collectively, these observations suggest that systematic ocular evaluation using OCT—encompassing both posterior segment parameters (RNFL, GCC, macular thickness) and anterior segment biometry (ACD, IOP)—may be considered in future comprehensive ophthalmologic screening protocols for obese children, particularly those with metabolic comorbidities such as insulin resistance, dyslipidemia, or elevated blood pressure, if confirmed by larger prospective studies. Prospective longitudinal studies are warranted to determine whether the subtle structural OCT changes observed in obese children progress over time and whether they predict future systemic or ocular complications.

Several limitations of this study warrant acknowledgment. First, the relatively small sample size (52 children, 104 eyes) limited statistical power, particularly for parameters with small-to-moderate effect sizes. A post hoc power analysis indicated that a minimum of 27–88 participants would be required to achieve 80% power for the primary parameters of interest (ACD and GCC average thickness), depending on the assumed effect size. Second, the retrospective cross-sectional design precluded causal inference, and the absence of baseline measurements limited the interpretability of group differences. Third, IOP was not systematically recorded, which may have confounded anterior segment findings. Fourth, pubertal status (Tanner stage) was not assessed; sex-related hormonal variation may have influenced certain ocular parameters independently of obesity. Fifth, the use of both eyes from each participant as analytical units in the descriptive comparisons is a methodological limitation, as fellow eyes are not fully independent. While cluster-robust bootstrap regression models—which treat the patient as the unit of inference—were used as the primary inferential analysis to address this, the eye-level descriptive *p*-values should be interpreted accordingly. Sixth, the high proportion of missing ACD values in the healthy control group (60.0%, 15/25 participants) represents a significant limitation. The complete-case ACD analysis was restricted to 10 healthy control participants with valid measurements, and the possibility of selection bias within the control group cannot be excluded. This substantially limits the interpretability of the ACD finding, which should be regarded as preliminary only. Seventh, the statistically significant age difference between groups (obese: 12.74 ± 3.04 years vs. healthy: 10.76 ± 3.03 years; *p* = 0.023) was addressed through covariate adjustment in regression models, but age-matching was not performed at study design, which constitutes a structural limitation. Eighth, the age- and sex-stratified subgroup analyses were highly underpowered, with some subgroups containing fewer than 10 participants. These analyses should be considered purely exploratory, and no biological conclusions should be drawn from them.

## 6. Conclusions

This study suggests that pediatric obesity may be associated with subtle changes in both retinal and anterior segment ocular structures. ACD showed an internally consistent exploratory pattern across unadjusted and adjusted analyses; however, it did not survive correction for multiple comparisons and should not be interpreted as a confirmatory finding. Because none of the investigated parameters remained statistically significant after Bonferroni correction, all findings must be considered exploratory and hypothesis-generating. Larger prospective, pre-registered studies with adequately powered cohorts are required to determine whether pediatric obesity is associated with structural ocular alterations over time.

## Data Availability

The data presented in this study are available from the corresponding author upon reasonable request.

## Abbreviations

The following abbreviations are used in this manuscript: OCT, optical coherence tomography; SD-OCT, spectral-domain optical coherence tomography; RNFL, retinal nerve fiber layer; GCC, ganglion cell complex; FLV, focal loss volume; GLV, global loss volume; EMM5, Early Treatment Macular Map 5; CCT, central corneal thickness; SimK, simulated keratometry; AL, axial length; ACD, anterior chamber depth; OLCR, optical low-coherence reflectometry; WTOW, white-to-white corneal diameter; BMI, body mass index; IOP, intraocular pressure; SE, spherical equivalent; CI, confidence interval.
